# Round the Hospitals

**Published:** 1918-04-27

**Authors:** 


### ROUND THE HOSPITALS.

WE are glad to have continuous evidence that certificated nurses are being stirred with interest and enthusiasm by the approaching elections to the Council of the College of Nursing, and the formation of local centres which the College is establishing generally throughout the country. Every-

☛ Previous articles appeared Jan. 19, 26; Feb. 9, 23; March 16; and April 6 and 20, p. 59.

**ROUND THE HOSPITALS—(*continued*).**

where keenness is shown by nurses to master the real objects of the College and its vital importance to the best interests of the nurses themselves. We welcome the demand for local centres, which has become a real living fact. The value to nurses of having an active branch of the College of this type within reach is exciting enthusiasm, and by the time of the annual meeting, fixed for June 6 next, local centres of the College should have multiplied. We are glad to note that the volume of work to be done, and the organising energy needed to expedite matters and give reality and force to each local centre, are fully recognised by the Council. At its last meeting they appointed Miss G. Cowlin to a new post, that of Organising Secretary. Miss Cowlin will hold a position of independence and be charged with the responsibility attaching to the formation of local centres and the active propaganda of the College. She has done yeoman service in connection with the Registration Department during the past year, and her new appointment will offer her more adequate scope for the marked talents of which experience has proved her to be the happy possessor.

---

The report and balance-sheet of the College for the year ending March 31, 1918, are in an active state of preparation. The statement of accounts with the balance-sheet have been audited, and will be shortly issued to the members. This report and the accounts which accompany it must have the widest interest for nurses who take pride in their profession. The accounts will no doubt attract close attention, seeing that they must demonstrate the business qualities of the College Council, and its claim to possess the complete confidence of certificated nurses throughout the country, and, indeed, the British Empire. The fixing of the date of the annual meeting so late as June 6 this year is a wise step, for it will enable the members to fill up the vacancies on the College Council and to master the report and accounts, which should place them in a position to show the keenness and ability they bring to bear on all matters affecting the welfare and development of their College, its organisation, and progress. The second annual meeting ought, therefore, to be a bumper so far as the number of attendances is concerned. Every member will no doubt note June 6 and keep it free wherever possible, so that she may attend and take part in the proceedings of the first assembly of members after the introduction of democratic government. Another point the members might prepare the way for is to further in their own interest the establishment of a local centre in their midst wherever their field of work may lie. This they might do by bringing a list of their sister nurses whom they have asked to sign a request to the Council to establish such a centre, which they might hand to the chairman at the annual meeting. By so doing they will add to their individual force, secure many facilities to cheer them in their work, promote unity and pleasant intercourse amongst their fellow-workers, and aid in the furtherance of developments calculated to help all nurses materially in the practice of their profession.

---

An important meeting, at which Sir William Cobbett will take the chair, will be held on Friday, April 26, at 4.30 p.m., in the out-patients' department of the Royal Infirmary, Manchester. The object is to bring before nurses the aims of the College of Nursing and to enlighten the public about the Nation's Tribute to Nurses. The Hon. Sir Arthur Stanley and Dame May Whitty will speak, and an influential gathering is anticipated. Other meetings of College centres this week at Leicester and at Leeds testify to the lively growth of that new plant, union among nurses.

---

The King has approved the award of the Military Medal to Staff Nurse Sarah Evelyn Johnson, Q.A.I.M.N.S.R., for gallantry, consistent good work, and devotion to duty. When the casualty clearing station was struck by a bomb from an aircraft she displayed great courage and coolness and set a splendid example to all by her absolute disregard of danger. Such deeds reflect back on the whole profession to which this brave lady belongs, and are a source of added strength and dignity to her sisters.

---

The new Nurses' Home at the London Hospital, which, by the express wish of Queen Alexandra, is to bear the name of Edith Cavell instead of Alexandra, will be opened early next month, and, so it is hoped, by Queen Alexandra in person. Sir George Frampton's bust of the heroic nurse, which is to be placed in the nurses' sitting-room, will be unveiled on this occasion. The London County Council had it in mind to place a memorial tablet to Edith Cavell on the London Hospital; but, having regard to the fact that the new Nurses' Home will bear her name in conspicuous letters for all who pass to see, they have practically decided to take no further steps in the matter.

---

Great anxiety has been felt regarding the safety of English nurses in Russia. The first British refugees from Moscow have now arrived in Yokohama. They number forty-five men and women, including Lady Muriel Paget, Sister Macdonald, Sister Cooke, Sister Davies, and Dr. Thompson, all belonging to the Anglo-Russian Hospital unit. No acts of hostility were committed against them, but they suffered much discomfort in the long, slow journey from South Russia. They are now comfortably housed in the old British naval hospital at Yokohama. There are still, we fear, many British nurses in Russia, but the fact that the above-named ladies have been in no way molested gives ground for hope that all may eventually reach this country in safety.

---

We quoted last week the statement of the Financial Secretary to the War Office to the effect that the sisters and nurses belonging to Q.A.I.M.N.S.R. are at present receiving pay and

**ROUND THE HOSPITALS—(continued).**

allowances as follows:—Sisters £50 to £65 per annum, and nurses £40 to £45, with an extra £20 a year if under engagement to remain as long as required, and a field allowance of 3s. a day, together with accommodation, fuel, and light. The messing allowance is 25s. a week, and uniform allowance £9 a year. It is interesting to note that the sisters of the Australian Nursing Service receive 9s. 6d. a day, or £173 a year, and their staff nurses 7s. a day, or £127 a year. Their messing allowance is 17s. 6d. a week, increased whenever necessary, and their uniform allowance is 10d. a day, or £15 a year. In the New Zealand Nursing Service sisters receive £120 a year, and staff nurses £75 to £100. The messing allowance is 24s. 6d. a week, with 4s. 8d. a week for laundry. The rate of pay commanded in civilian hospitals in the Dominions is considerably higher than in this country, and especially is this the case in regard to the higher ranks of the profession. There must be a great levelling-up process in this country before matrons and sisters touch the same rate of pay as overseas. So long as each Nursing Service is working in its own country the difference in pay is less marked, since the purchasing value of a sovereign is greater in this country than in Australia or New Zealand. It is when nurses from these different Services are working together in France or Egypt that such distinctions in pay are forcibly obtruded and arouse comment.

A writer in the *Red Cross Journal* this month makes the erroneous assertion that "our Dominions' Nursing Services permit dancing." On the contrary, the Matrons-in-Chief both of the Australian and the New Zealand Nursing Services have wisely decreed that the regulations deemed necessary for British sisters and nurses in war-time shall be binding on their own nurses. They are fully alive to the reasons which have actuated the home nursing authorities in making certain restrictions, and we believe also there are very few nurses indeed who desire them removed. Sitting at home in quiet localities, untouched by the mighty disintegrating force of war, people may deem it unreasonable to grudge the nurses "a little harmless recreation." This is the way it is put. But those at the helm are better judges of the sunken rocks. On them the responsibility rests, and they have abundantly proved themselves worthy of the trust. It is not in this direction that our cousins from overseas are disposed to criticise or suggest innovations, and their warm-hearted loyalty to the heads of the British Military Nursing Services is the greatest possible support in this time of crisis.

The remarkable change which has passed over the Poor-Law infirmaries of London during recent years is strikingly exemplified by the stream of children of all ages which pours into their wards. At the Kensington Infirmary at the present time there are no fewer than 160 children suffering from complaints which require skilled medical and nursing care. In one ward were six little pneumonia tents, and if ever there was a time when the word "infirmary" suggested none but chronic cases it is not now. The change which has passed over the spirit of the training-school under these circumstances may be imagined. The recently appointed medical superintendent, Dr. Remington Hobbs, has in view the reconstruction of the operating-room and an instalment of *x*-rays, a change which will be of immense advantage to the probationers. The nursing staff now numbers seventy, having been increased by twenty-three during the term of office of Miss Alsop, the present matron. The training is for three years, but a fourth year is available for specially selected nurses after obtaining their certificates, and gives them the privilege of midwifery and massage training in addition. Some fifteen nurses complete their training yearly, and of these nearly all now offer their services for war nursing. Lately orders have been received for the preparation of two wards, containing forty beds each, for the reception of the wounded.

It must always be true that efficient training depends primarily on the character of the clinical opportunities which can be offered to the nurses. But the quality of the probationers attracted to particular institutions is largely influenced by the quarters in which they are housed. The Kensington Infirmary is too good in other important respects to rest long content with the thoroughly inadequate nature of the nurses' rooms. All the probationers ought to be got out of cubicles into cheerful single bedrooms, and there should be a more light and suitable sitting-room for the nurses; that of the sisters is good. Want of space is an excuse which always yields to brains plus determination. The richest quarter in London ought not to permit its municipal nurses to be despicably housed.

The Church of St. Elizabeth, to which the Rev. Albert Lombardini was appointed as Chaplain of the Kensington Infirmary in 1912, has become not merely a centre of religious light to the staff and inmates of this great institution, but also a place of much artistic interest to the public. Built when the infirmary was founded last century, the chapel had grown gloomy and, truth to tell, neglected in appearance, until the present Chaplain, perceiving the scope it afforded for decoration on its large wall space, began to make its needs known. Some £2,000 has been spent already, and the interior begins to glow with light and colour. The memorial panels, designed and executed by Mr. William Glazby, are particularly effective. The great treasure is Mr. Frank Brangwyn's celebrated picture "Mater Dolorosa Belgica," representing a bruised and broken warrior, supported by his mother, who raises his hand to her lips. It is symbolical of Belgium in the hour of its great tragedy. But the picture in its present environment takes on another colour of symbolism, and seems to typify those lives broken in the battle of ruthless competition which find succour and sup-

**ROUND THE HOSPITALS**—(*continued*).

port within these walls in the hour when all is lost. The patients take a vivid interest in the adornment of their chapel, and linger admiringly among its beauties. They are to some extent infirm, blind, chronically ailing folk, depressing to work among, some might think. But their Chaplain thinks otherwise, and tells us: "The fire is always there, and when it is tended it glows."

The Hendon Infirmary was built not many years ago to receive chronic and infirm cases from the Westminster Union, and is a model of construction, perfectly adapted to its ends. Considerable difficulty has, however, been experienced from time to time in procuring for the nurses in the training-school adequate opportunities. There are practically no acute or surgical cases. The probationer nurses recently presented a petition to the infirmary committee setting forth these facts and others relating to their scale of remuneration and work. The facts have been confirmed by the medical superintendent, and the infirmary committee have reached the conclusion that the infirmary ought no longer to be maintained as a general training-school for nurses. Accordingly they have formulated a scheme under which the Union Infirmary at Hendon will be converted, as soon as the necessary alterations can be carried out, into an infirmary for the treatment of tuberculosis, the chronic and aged cases being removed to the Fulham Road Workhouse. Although there was some opposition to this report it was ultimately adopted. It is one that does honour to the committee. The weight attached by them to the question of giving adequate training to the probationer nurses illustrates the great advance which has been made of recent years in this direction. The decision to make these radical alterations has been arrived at from the point of view of good nursing administration, which is clearly seen to run parallel with the welfare of the patients.

Princess Beatrice met with a very cordial reception on the occasion of her visit on April 22 to the Imperial Nurses' Club, of which she is President. She was received by the Chairman of the Club Committee, Mrs. William Scott, and by the Honorary Secretary, Miss C. H. Mayers. Among those members staying in the Club at the time who had the honour of being presented to Her Royal Highness were Miss Bickerton, matron of the Prince of Wales's Hospital; Miss Burgess, R.R.C., matron of Crumpsall Infirmary; Miss Dann, R.R.C.; Miss H. T. Husband, matron of Maudsley Hospital, S.E.; Miss Ross, R.R.C., matron of the Hope Hospital, Manchester; Miss E. M. Smith, R.R.C., matron of No. 11 Military Hospital, Manchester; and Sister Mary Kennedy, R.R.C. Two Spanish nurses were also presented, their Queen being the daughter of the President, who was greatly interested to find them in the Club. The Princess made a thorough inspection of the premises, including the new annexe, for which a considerable outlay has been incurred necessitating an increase of capital. Further extension is in contemplation. One of the new rooms is named after Princess Beatrice, and for this she has kindly presented her portrait. During the past year, the first of the Club's existence, 1,690 nurses have slept in it, and it has been visited by nurses from Australia, America, Canada, South Africa, New Zealand, France, Switzerland, Italy, Norway, and Sweden. The yearly subscription for trained nurses is 10s. 6d., and for nurses in training 7s. 6d.

In New York a movement has been started, we learn from the *American Journal of Nursing*, to organise the services of married and retired nurses, in order to minimise the dislocation of the public services caused by the withdrawal of so many nurses for military purposes. A central committee has been formed with a chairman, under whom are sub-chairmen, each of whom has charge of a group of twelve nurses, enrolled to give definite service of some kind in case of immediate need. The training-schools to which they formerly belonged are giving these volunteers "brush-up work in clinics, social service work, operating-rooms," etc. It is believed these nurses, none of whom can give whole time, will be able to undertake the relief of tired nurses on special duty; the relief for hours or half-days of pupils, general duty nurses, registrars; the assistance of superintendents of nurses with clerical work, filing, and correspondence; assistance in operating-rooms by cleaning instruments or utensils and by making dressings; nursing treatment by the hour to patients unable to find resident nurses; classes to nurses in training-schools, and the conduct of Red Cross classes of instruction for volunteers. We are confident that the services of the married and retired nurses in London have never yet been fully utilised, and that much more might be done to bring them into line in the nation's hour of need.

The need for better administration in district nursing and child-welfare work in Melbourne is exemplified by the application of the Visiting Nurses' Association to the State Treasurer for an allocation of £500 with which to supplement the earnings of nurses in the poorer suburbs. The Treasurer suggested that the earnings of all the visiting nurses be pooled so that receipts from the more affluent suburbs might balance the scale. On the face of it this sounds as unfair as if it were proposed to pool the earnings of all the doctors or all the architects. If anything of the kind be attempted, something in the nature of a municipal Nursing Service would be a more equitable plan. Each visiting nurse should be guaranteed a good salary, and the fees should be paid into a central fund. Again, Cabinet Ministers are protesting against the exaggerated cost of the existing child-welfare schemes, and public aid appears to be given or withheld, without any continuity of policy, to such needful undertakings as the supply of pure milk to the babies. If Melbourne is to set the pace for the Old Country it must do better than this.

☞ For Letter on "The Employment of the Waiting Years," see p. 79; Nursing Affairs in Ireland, p. 80; Matrons' and Sisters' Appointments, p. 85.

